# Clinical evaluation of intensity-modulated radiotherapy for locally advanced pancreatic cancer

**DOI:** 10.1186/s13014-018-1063-5

**Published:** 2018-06-25

**Authors:** Yoko Goto, Akira Nakamura, Ryo Ashida, Katsuyuki Sakanaka, Satoshi Itasaka, Keiko Shibuya, Shigemi Matsumoto, Masashi Kanai, Hiroyoshi Isoda, Toshihiko Masui, Yuzo Kodama, Kyoichi Takaori, Masahiro Hiraoka, Takashi Mizowaki

**Affiliations:** 1 Department of Radiation Oncology and Image-applied Therapy, Kyoto University Graduate School of Medicine, 54 Shogoin, Kawaracho, Sakyo-ku, Kyoto, Japan; 2 Department of Radiation Oncology, Graduate School of Medicine, Yamaguchi University, Yamaguchi, Japan; 3 Department of Clinical Oncology, Kyoto University Graduate School of Medicine, Kyoto, Japan; 4 Department of Diagnostic Imaging and Nuclear Medicine, Kyoto University Graduate School of Medicine, Kyoto, Japan; 5 Department of Surgery, Kyoto University Graduate School of Medicine, Kyoto, Japan; 6 Department of Gastroenterology and Hepatology, Kyoto University Graduate School of Medicine, Kyoto, Japan; 7 Department of Radiation Oncology, Japanese Red Cross Society Wakayama Medical Center, Wakayama, Japan

**Keywords:** Intensity-modulated radiotherapy, Chemoradiotherapy, Locally advanced pancreatic cancer, Treatment result

## Abstract

**Background:**

The purpose was to retrospectively evaluate the effect of intensity-modulated radiotherapy (IMRT) on gastrointestinal (GI) toxicities and outcomes compared to three-dimensional conformal radiotherapy (3DCRT) for locally advanced pancreatic cancer (LAPC).

**Methods:**

We included 107 consecutive patients who underwent CRT for LAPC from September 2001 to March 2015; 80 patients underwent 3DCRT and 27 patients underwent IMRT. They were compared for GI toxicities, locoregional progression free survival (LRPFS), distant metastasis free survival (DMS), and overall survival (OS).

**Results:**

Median radiation dose and fractions for 3DCRT and IMRT were 54 Gy/30 fr. and 48 Gy/15 fr. The regimens of CRT consisted of weekly gemcitabine 250 mg/m^2^ (for 3DCRT) or 1000 mg/m^2^ (for IMRT). Acute GI toxicity ≥grade 2 occurred in 32 patients (40%) treated with 3DCRT compared with five patients (19%) treated with IMRT. Late GI toxicity of grade 3 occurred in 10 patients (12%) treated with 3DCRT and one patient (4%) treated with IMRT. Patients who underwent IMRT had superior 1-year LRPFS (73.1% vs. 63.2%, *p* = 0.035) and 1-year OS (92.3% vs. 68.2%, *p* = 0.037) as compared with those treated with 3DCRT. Multivariate analysis showed that in IMRT patients, higher dose (≥45 Gy) was an independent factor for better LRPFS and OS.

**Conclusions:**

LAPC patients treated with hypofractionated full-dose gemcitabine IMRT had improved OS and LRPFS without increased GI toxicities when compared to those of patients treated with conventionally fractionated low dose gemcitabine 3DCRT. In IMRT patients, higher dose was an independent favorable prognostic factor for better LRPFS and OS, which suggests that dose escalation with IMRT for LAPC is a promising strategy.

## Background

Pancreatic cancer is fatal for most patients, and it is the fourth leading cause of death from malignancies in Japan. Only surgical resection offers a potentially curative approach, but merely 5 to 25% of patients present with resectable disease [1, 2]. Approximately 35% of patients with pancreatic cancer have unresectable locally advanced pancreatic cancer (LAPC), and the treatment for them is chemotherapy with or without radiotherapy [3]. The prognosis of LAPC patients has been poor, with a median survival of 9 to 13 months and 5-year overall survival (OS) of < 5%. Distant metastases are a dominant cause of disease progression. However, a recent autopsy study revealed that about one-third of patients with pancreatic cancer die from locally destructive disease rather than distant metastasis [4]. This suggests that local control is meaningful to prevent tumor progression and improved survival of LAPC patients.

For several decades, the role of radiotherapy for LAPC remained controversial. Since the 1980s, several randomized control trials of LAPC patients comparing chemoradiotherapy (CRT) with chemotherapy were conducted [5–7]. However, the results were contradictory. The results of a recently published randomized LAP07 trial demonstrated no significant survival benefits with the addition of radiotherapy to chemotherapy for LAPC patients [8]. In addition, more intensive regimens of chemotherapy, such as FOLFIRINOX and gemcitabine plus nab-paclitaxel, are reported to improve survival of metastatic pancreatic cancer patients, and are expected to be effective for LAPC patients [9–11]. This raises serious questions about the role of radiotherapy in the management of LAPC patients.

The biggest problem with CRT for pancreatic cancer is that tumor is surrounded by radiosensitive gastrointestinal (GI) organs, such as the stomach and the duodenum. This anatomical situation makes it difficult to deliver high doses to tumor without increasing irradiation dose to GI organs. The severe GI toxicity is related with the irradiated volume and dose received on the stomach and duodenum [12]. With the introduction of intensity-modulated radiotherapy (IMRT) which can simultaneously reduce the dose to surrounding normal organs, while allowing an increase in target tumor dose, it is expected that the rate of GI toxicities will be reduced and the efficacy may be increased [13, 14].

In our institution, we have been performing IMRT for LAPC since 2009. Before IMRT, we treated LAPC patients with 3DCRT at a total dose of 54 Gy/30 fr. with weekly gemcitabine at 250 mg/m^2^ based on the results of phase I and phase II trials in our institution [15, 16]. The treatment regimen was well-tolerated and provided prolonged survival in LAPC patients; the 1-year survival rate was 74% and the MST was 16.6 months. However, the rate of distant metastasis was high. When we introduced IMRT for LAPC, we decided to use full dose gemcitabine in combination with radiotherapy and shorten radiation fractionation from thirty to fifteen, based on a previous report [17]. We conducted phase I dose escalation study to determine the maximum tolerated radiation dose delivered by IMRT with full-dose gemcitabine, and it was 48 Gy/15 fr., (biological equivalent dose (BED_10_) = 63.4Gy, UMIN000004589). In this study, our aim is to retrospectively evaluate the effect of IMRT on outcomes and treatment-related acute and late GI toxicities compared with 3DCRT for LAPC.

## Methods

### Patient characteristics

The clinical data of LAPC patients treated with definitive CRT from September 2001 to March 2015 at our institution were retrospectively reviewed. Locally advanced unresectable disease was defined as superior mesenteric artery or celiac axis encasement > 180 degrees, unreconstructible superior mesenteric vein/portal occlusion, or aortic invasion. Patient, tumor, and treatment characteristics were obtained from the medical records.

All patients were monitored weekly during CRT for acute GI toxicities, including nausea, vomiting, diarrhea, and abdominal pain. After CRT ended, patients were followed up once every 2 months, and monitored for late GI toxicities, including gastroduodenal ulcer and hemorrhage. All toxicities were scored according to the Common Terminology Criteria for Adverse Events (CTCAE), version 4.0. The institutional Review Board of Kyoto University Hospital approved this study.

### Chemotherapy

The regimen of induction chemotherapy consisted of weekly intravenous administration of 1000 mg/m^2^ of gemcitabine on days 1, 8, 15 during 4-weeks. The regimens of CRT consisted of weekly gemcitabine 250 mg/m^2^ (for 3DCRT) or 1000 mg/m^2^ (for IMRT). As additional treatment after radiotherapy, 3 weekly doses of gemcitabine at 1000 mg/m^2^ every 28 days were administered until the tumor progression or patient refusal. If patients had some complications associated with gemcitabine, such as intestinal pneumonia, 80 mg/m^2^/day of S-1 was administered orally during radiotherapy twice daily on weekdays. After first-line gemcitabine-based therapy, S-1 based chemotherapies are the most frequently used, although this was left to the discretion of the medical oncologist.

### Radiotherapy

Treatment-planning computed tomography (CT) was performed with intravenous contrast media, without oral contrast agents. The gross target volume (GTV) included radiographically apparent gross tumor and suspicious/enlarged lymph nodes on CT simulation for 3DCRT plans. The clinical target volume (CTV) was defined as the GTV plus a 5-mm margin in all directions. The CTV also included the potential para-aortic lymph node and neuroplexus involvement between the celiac axis and the superior mesenteric artery. The planned target volume (PTV) was determined by adding a horizontal 5-mm margin and a cephalocaudal 10-mm margin to the CTV, considering respiratory movement. A total dose of 54 Gy was delivered in 30 fractions, using 3- or 4-field planning and a dynamic arc conformal technique.

For IMRT plans, the GTV and CTV were the same as those of 3DCRT plans. The PTV was generated by adding a 5-mm margin in all directions to the CTV in consideration of respiratory management. For IMRT, breath-hold method or tumor dynamic tracking method were adopted for the management of tumor respiratory motion. IMRT planning was performed, using a commercially available planning system (Eclipse™ Varian, Medical Systems, Palo Alto, CA). The prescription dose was specified as D95 (the dose that covers 95% of the structure) to PTV-boost, a volume that subtracted normal organs (the stomach and the duodenum) plus 5- to 10-mm margins from PTV. The prescription dose was individualized between 39 and 51 Gy/15 fr. by achieving the dose constrain for OARs and referring to a previous institutional trial (UMIN000004589). The dose constraints of the OARs are listed in Table 1. The standard beam arrangement involved five to six gantry angles. Treatment was delivered using dynamic multileaf collimation.

**Table 1:** Dose constraints for OAR

Structure	Constraints
Stomach/Duodenum	V45 Gy < 1 cc
	V42 Gy < 5 cc
	V39 Gy < 25 cc
Stomach + PRV/Duodenum + PRV	V39 Gy < 30 cc
	V36 Gy < 45 cc
Spinal cord	Dmax < 36 Gy
Spinal cord + PRV	D2 cc < 39 Gy
Kidney (at least one)	V20 Gy < 30%
Liver	Dmean < 30 Gy

*Abbreviations*; *OAR* organs at risk, *PRV* planning organ at risk volume, *Dmax* he maximum dose to the structure volume, *Dmean* the mean dose to the structure volume, *D2 cc* the maximum dose covering ≥2 cc of the structure volume, *VxxGy* the volume of the structure receiving > xx Gy

### Statistics

Fisher exact test was performed to compare the characteristics of patients treated with 3DCRT versus those treated with IMRT. Overall survival (OS) was defined as the period from the chemotherapy or chemoradiotherapy starting date to the date of death of any cause, and it was censored at the last follow-up visit for living patients. The Kaplan-Meier method was used to estimate the OS, LRPFS, and DMFS. The log-rank test was performed for the OS, LRPFS, and DMFS comparisons. The Cox proportional hazard model was used to estimate the hazard ratio. The chi-squared test was used to compare the rates of acute GI toxicities and the Gray’s test was used to compare the cumulative incidence rates of late GI toxicities of patients treated with 3DCRT versus those with IMRT. All statistical tests were 2-sided. The difference was deemed statistically significant when the *p* value was <.05. All statistical analyses were performed using EZR version 1.11 (Saitama Medical Center, Jichi Medical University, Saitama, Japan), which is a graphical user interface for R version 2.13.2 (The R Foundation for Statistical Computing, Vienna, Austria).

## Results

### Patient and tumor characteristics

In total, 107 consecutive patients who underwent CRT for LAPC from September 2001 to March 2015 were included in this analysis; 80 patients were treated with 3DCRT (75%), and 27 patients with IMRT (25%). Of the 107 patients, 58% were male, and the median age at diagnosis was 65 years old (range, 35–85 years old). Fifty-three percent of tumors were located in the head/neck of the pancreas and 47% in the body/tail.

Sixty-three (59%) patients received induction chemotherapy, administered for 1–12 months prior to radiotherapy. In our protocol of IMRT clinical trial, the induction chemotherapy is one course of weekly administration of gemcitabine on days 1, 8, 15 during 4-weeks. All patients received concurrent chemotherapy during radiotherapy. Most patients (93%) received gemcitabine, 8 patients (7%) received it with S-1. Median radiation doses and fractions for 3DCRT and IMRT were 54 Gy/30 fr. (range, 48.6–55.6 Gy) and 48 Gy/15 fr. (range, 39–51 Gy), respectively. Patient, tumor, and treatment characteristics are summarized in Table 2.

**Table 2:** Patient characteristics of tumors and treatment

Characteristic	3DCRT	IMRT	*p*-value
Number of patients (n)	80	27	
Age (median, range)	65, 35–85	66, 42–84	0.57
Gender (male/female)	45/35	10/17	0.65
PS (0–1/2)	74/6	27/0	0.33
Tumor location(head, uncus/body, tail)	41/39	16/10	0.51
Tumor size (median, range [mm])	29, 19–40	30, 10–70	0.038
Induction chemotherapy (yes/no)	36/44	27/0	< 0.01
Concurrent chemotherapy (Gemcitabine/S-1)	73/7	26/1	0.68
Radiation dose (median, range [Gy])	54, 48.6–55.8	48, 39–51	
Radiation fractionation (median, range)	30, 27–31	15, 15–15	

*Abbreviations*; *PS* performance status

### Treatment outcome

**Fig. 1:**
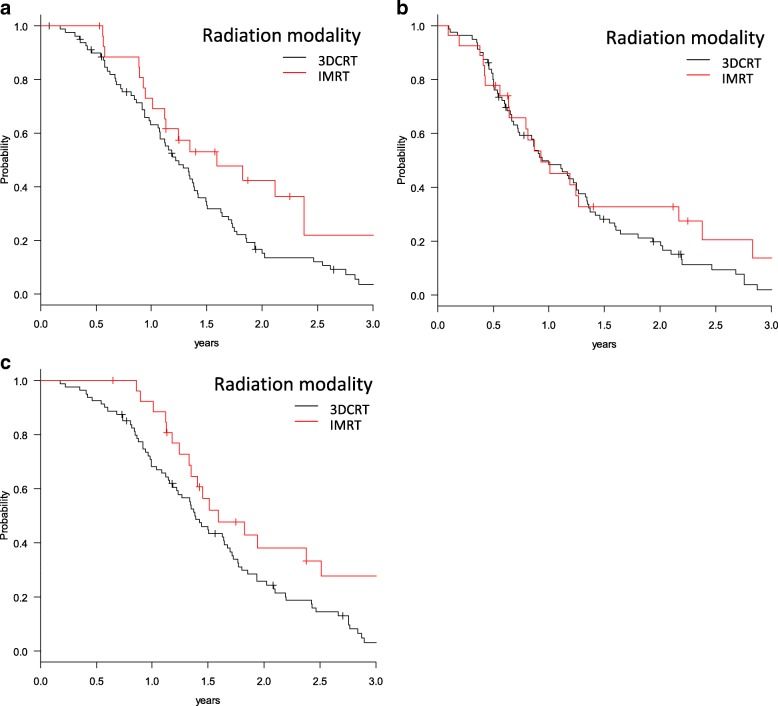
Kaplan-Meier estimates of (**a**) Locoregional progression free survival (LRPFS), (**b**) Distant metastasis free survival (DMFS) and (**c**) overall survival (OS) by radiation modality of patients who received 3DCRT (*n* = 80) and IMRT (*n* = 27)

Median follow-up period was 16.4 months, which was similar between groups. The 1-year OS rate of all patients was 74.3%, and median survival time (MST) was 17.5 months. The 1-year OS rates in the 3DCRT and IMRT groups were 68.2 and 92.3%, respectively (*p* = 0.0369). The 1-year LRPFS rates in the 3DCRT and IMRT groups were 63.2 and 73.1%, respectively (*p* = 0.0349). The 1-year DMFS rates in the 3DCRT and IMRT groups were 48.4 and 49.3%, respectively (*p* = 0.308) (Fig. 1).

### Univariate and multivariate analyses in all patients

The results of univariate analyses for LRPFS and OS are shown in Table 3. Radiation modality of 3DCRT (*p* = 0.037), larger tumor (*p* = 0.047), and high pretreatment CA19–9 level (*p* = 0.010) were found to be significant unfavorable factors for OS. Younger age (*p* = 0.039), radiation modality of 3DCRT (*p* = 0.035), and high pretreatment CA19–9 level (*p* < 0.01) were significant unfavorable factors for LRPFS.

**Table 3:** Univariate analysis of all patients

Factors	Number of patients	1-year LRPFS (%)	*p*-value	1-year OS (%)	*p*-value
Age (years)					
< 66	58	52.6	0.039	64.7	0.11
≥66	49	81.0		85.5	
Gender					
Male	45	71.9	0.88	77.4	0.67
Female	62	59.5		72.0	
Tumor size					
< 3 cm	64	65.0	0.11	77.7	0.047
≥3 cm	43	66.7		69.3	
Radiation modality					
3DCRT	80	63.2	0.035	68.2	0.037
IMRT	27	73.1		92.3	
PS					
0	52	65.2	0.55	78.3	0.053
1–2	55	66.2		70.5	
Pretreatment CA19–9 (U/ml)					
< 300	64	76.7	0.0026	82.2	0.010
≥300	43	50.0		62.8	
Tumor location					
Body/Tail	50	69.4	0.24	76.0	0.21
Head/Neck	57	62.3		72.8	

*Abbreviations; LRPFS* locoregional progression free survival, *OS* overall survival

The results of multivariate analysis for OS are listed in Table 4. Multivariate analyses revealed that high pretreatment CA19–9 level (*p* = 0.026) was a significant unfavorable factor and radiation modality of 3DCRT (*p* = 0.082) was a marginal unfavorable factor.

**Table 4:** Multivariate analysis of all patients

Factors	Number of patients	OS	
		HR (95% CI)	*p*-value
Radiation modality			
3DCRT	80		
IMRT	27	0.64 (0.38–1.06)	0.082
Pretreatment CA19–9 (U/ml)			
< 300	64		
≥300	43	1.62 (1.06–2.47)	0.026
Tumor size			
< 3 cm	64		
≥3 cm	43	1.37 (0.89–2.10)	0.15

*Abbreviations*; *OS* overall survival, *HR* hazard ratio

### Univariate and multivariate analyses in patients treated with IMRT

The results of univariate analyses for LRPFS and OA are shown in Table 5. Radiation dose under 45Gy (*p* < 0.01) and high pretreatment CA19–9 level (p = 0.037) were found to be significant unfavorable factors for OS. Younger age (*p* = 0.015), radiation dose under 45Gy (p < 0.01), and high pretreatment CA19–9 level (*p* = 0.032) were significant unfavorable factors for LRPFS. As younger age was a significant unfavorable factor, it would be important to observe younger patients closely post-CRT.

**Table 5:** Univariate analysis of IMRT patients

Factors	Number of patients	1-year LRPFS (%)	*p*-value	1-year OS (%)	*p*-value
Age (years)					
< 66	12	50	0.015	83.3	0.21
≥66	15	92.9		100	
Gender					
Male	17	68.8	0.63	93.8	0.72
Female	10	80		90	
Tumor size					
< 3 cm	19	72.2	0.36	94.4	0.23
≥3 cm	8	75		87.5	
Radiation dose					
< 45 Gy	9	33.3	1.23E-07	88.9	0.000077
≥45 Gy	18	94.1		94.1	
PS					
0	15	60	0.41	86.7	0.47
1	12	90.9		100	
Pretreatment CA19–9 (U/ml)					
< 300	17	87.5	0.032	100	0.037
≥300	10	50		80	
Tumor location					
Body/Tail	11	54.5	0.25	81.8	0.12
Head/Neck	16	86.7		100	

*Abbreviations*; *LRPFS* locoregional progression free survival, *OS* overall survival

**Fig. 2:**
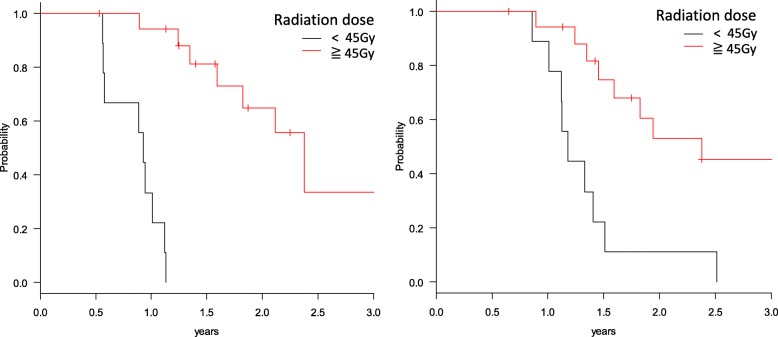
Kaplan-Meier estimates of (**a**) Locoregional progression free survival (LRPFS) and (**b**) overall survival (OS), according to total dose ≥45 Gy or < 45 Gy

The results of multivariate analyses for LRPFS and OA are listed in Table 6. Multivariate analyses revealed that radiation dose under 45Gy was a significant unfavorable factor for both LRPFS (p < 0.01) and OS (*p* = 0.044). Eighteen patients in the IMRT group who were treated with ≥45 Gy had significantly better 1-year LRPFS (94.1% vs. 33.3%, p < 0.01) and 1-year OS (94.1% vs. 88.9%, p < 0.01) compared with IMRT patients treated with < 45 Gy (Fig. 2). There was no significant difference of tumor sizes among IMRT patients who had been treated with under 45Gy and over 45Gy (31.4 mm vs. 27.2 mm, *p* = 0.18). The MST of patients treated with IMRT over 45Gy and under 45Gy were 28.8 months and 14.3 months, respectively.

**Table 6:** Multivariate analysis of IMRT patients

Factors	Number of patients	LRPFS		OS	
		HR (95% CI)	*p*-value	HR (95% CI)	*p*-value
Age (years)					
< 66	12				
≥66	15	0.23 (0.064–0.86)	0.029	0.86 (0.28–2.67)	0.79
Radiation dose					
< 45 Gy	9				
≥45 Gy	18	0.043 (0.0049–0.38)	4.00E-03	0.27 (0.077–0.96)	0.044
Pretreatment CA19–9 (U/ml)					
< 300	17				
≥300	10	3.39 (0.85–13.54)	0.084	1.87 (0.63–5.55)	0.26
Tumor location					
Body/Tail	11				
Head/Neck	16	0.73(0.21–2.58)	0.62	0.61 (0.20–1.9)	0.39

*Abbreviations; LRPFS* locoregional progression free survival, *OS* overall survival, *HR* hazard ratio

### Recurrence pattern

Among the 27 patients who underwent IMRT, the first relapse occurred at locoregional in 3 patients (11.1%) and at distant organs in 16 patients (59.3%), and at both locoregional and distant organs in 1 patient (3.7%). Among the 80 patients who underwent 3DCRT, the first relapse occurred at locoregional in 24 patients (30%) and at distant organs in 42 patients (52.5%), and at both locoregional and distant organs in 4 patients (5%).

### GI toxicities

An overview of the acute and late GI toxicities and their grades is shown in Table 7. Acute GI toxicity of grade 2 or higher occurred in 32 patients (40%) treated with 3DCRT compared with five patients (19%) treated with IMRT (*p* = 0.042). Late GI toxicity of grade 2 or higher occurred in 10 patients (12%) treated with 3DCRT compared with two patients (8%) treated with IMRT. As for late GI toxicity of grade 2 or higher, grade 3 toxicities occurred in 10 patients (12%) treated with 3DCRT and one patient (4%) treated with IMRT, respectively. GI toxicity of grade 4 or higher was not observed. The 1-year cumulative incidence rates of late GI toxicities of grade 2 or higher in the 3DCRT and IMRT groups were 11.3% (95% CI = 5.5–19.4%) and 7.6% (95% CI = 1.3–21.7%), respectively (*p* = 0.47).

**Table 7:** Acute and late gastrointestinal toxicity

	Gr 0–1	Gr 2	Gr 3	Gr 4
Acute GI toxicity				
3D-CRT	48 (60%)	29 (36%)	3 (4%)	0 (0%)
IMRT	22 (81%)	5 (19%)	0 (0%)	0 (0%)
Late GI toxicity				
3DCRT	70 (88%)	0 (0%)	10 (12%)	0 (0%)
IMRT	25 (92%)	1 (4%)	1 (4%)	0 (0%)

*Abbreviations; GI* gastrointestinal

## Discussion

The present study aimed to evaluate the effect of hypofractionated full-dose gemcitabine IMRT on GI toxicities and outcomes compared to conventionally fractionated low dose gemcitabine 3DCRT for LAPC patients.

Pancreas is surrounded by radiosensitive GI organs such as the stomach and the duodenum, and this anatomical situation makes it difficult to deliver high doses to tumor without increasing the irradiation dose to GI organs^12,13^. A previous attempt for dose-escalation when performing 3DCRT resulted in failure in LAPC; it was more toxic and less effective [6]. Because IMRT can deliver high-dose radiation to the target volume while decreasing the radiation to dose-limiting adjacent critical structures, it has been suggested to be beneficial for LAPC. A systematic review showed that GI toxicities were significantly reduced with IMRT [18]. Similar to the previous reports, the rates of GI toxicities were low in our study; only 4 % of IMRT patients experienced grade 3+ late GI toxicity, compared with 12% of 3DCRT patients. In addition, concerning acute hematological toxicities in the IMRT patients, grade 3 leukopenia, neutropenia and thrombocytopenia occurred in 10, 7, and 1 patients, respectively. Grade 4 neutropenia occurred in 3 patients (11%); however, all acute hematologic toxicities were able to be well managed. IMRT enabled us to use full dose gemcitabine in combination with radiotherapy of shorten fractionation without increasing GI toxicities when compared to 3DCRT.

This study revealed that patients treated with IMRT showed significantly improved LRPFS and OS compared with those treated with 3DCRT, whereas there was no significant difference in DMFS between the two groups. This suggests that better locoregional control would result in better OS. Several dose escalation studies, using IMRT technique reported hopeful results. Ben-josef et al. conducted a phase I/II trial of IMRT dose escalation in LAPC patients [19]. They reported that high-dose radiation therapy, 55 Gy/25 fr. can be administered safely with concurrent full-dose gemcitabine, and the median OS after such therapy was 15 months. Recently, Krishnan et al. reported that dose escalated IMRT (BED > 70 Gy) for the patients who had tumors > 1-cm from the luminal organs is feasible and tolerable [20]. They reported that higher dose (BED) was a strong independent predictor of improved OS in those patients. In our cohort, the patients who received over 45 Gy, using IMRT had significantly better OS than those receiving under 42 Gy of IMRT (MST 28.8 months vs. 14.3 months, *p* < 0.01), which also suggests that dose escalation with IMRT for LAPC is a promising strategy.

As for recurrence pattern, patients who underwent IMRT had less locoregional recurrence as a first site compared with those who underwent 3DCRT (11.1% vs. 30%). Locoregional relapse is often associated with pain, gastroduodenal obstruction. Therefore, improved locoregional control would be beneficial for keeping quality of life for the patients of LAPC. On the other hand, the rate of distant metastasis as a first site is still high which is approximately 50–60%. This suggest that more efficient systemic chemotherapy is needed to treat micrometastatic spread in these patients. Low toxicity of CRT using IMRT will make it possible to receive further treatment with intensive systemic chemotherapies, and FOLFIRINOX or gemcitabine plus nab-paclitaxel regimens before or after CRT may address these issues better [10, 11].

As with any retrospective analysis, there are several limitations. First, the assessment of toxicities in a retrospective analysis tends to underestimate risks owing to incomplete recording of side effects and the recall bias. However, severe toxicities would have required additional medical care, which would have been clearly documented. Second, IMRT was administered as part of an institutional change in practice in 2009, rather than in a prospective controlled manner. In addition, the dose of concurrent chemotherapy and the rate of patients who received induction chemotherapy were different between the 3DCRT and IMRT patients, and respiratory management was used in IMRT patients only. However, all treatment plans were developed and conducted at one institution, limiting the bias introduced by multi-institutional plans. Third, this retrospective analysis did not directly compare the outcomes between CRT and chemotherapy for LAPC patients. Clinical studies, including the LAP07 randomized clinical trial, which could not show survival benefit of CRT over chemotherapy alone for LAPC adopted the conventional radiotherapy technique, 3DCRT [6, 8]. In this study, the MST of the patients treated with IMRT under 45 Gy was 14.3 months, which was approximately equal to the results of CRT or chemotherapy patients in LAP07 trial. On the other hand, the MST of the patients treated with IMRT ≥45 Gy was 28.8 months, and it is much better than the result of the patients treated with IMRT under 45 Gy. This result prompted us to verify the role of radiotherapy, especially IMRT, for LAPC patients.

In summary, our data demonstrated that LAPC patients treated with hypofractionated full-dose gemcitabine IMRT had improved OS and LRPFS without increased GI toxicities when compared to conventionally fractionated low dose gemcitabine 3DCRT, suggesting that intensified CRT, using IMRT might be beneficial for LAPC patients. To evaluate this prospectively, we are currently conducting phase II multi-institutional clinical trial of CRT, using IMRT for LAPC patients (UMIN000017521).

## Conclusions

LAPC patients treated with hypofractionated full-dose gemcitabine IMRT had improved OS and LRPFS without increased GI toxicities when compared to those of patients treated with conventionally fractionated low dose gemcitabine 3DCRT. In IMRT patients, higher dose was an independent favorable prognostic factor for better LRPFS and OS, which suggests that dose escalation with IMRT for LAPC is a promising strategy.

## Abbreviations

3DCRT: three-dimensional conformal radiotherapy
BED: biological equivalent dose
CRT: chemoradiotherapy
CTCAE: Common Terminology Criteria for Adverse Events
CTV: clinical target volume
DMS: distant metastasis free survival
GI: gastrointestinal
GTV: gross tumor volume
IMRT: intensity-modulated radiotherapy
LAPC: locally advanced pancreatic cancer
LRPFS: locoregional progression free survival
MST: median survival time
OS: overall survival
PTV: planned target volume

## References

[1] Willett CG, Czito BG, Bendell JC, Ryan DP (2005). Locally advanced pancreatic cancer. Journal of clinical oncology : official journal of the American Society of Clinical Oncology..

[2] Krishnan S, Rana V, Janjan NA, Varadhachary GR, Abbruzzese JL, Das P (2007). Induction chemotherapy selects patients with locally advanced, unresectable pancreatic cancer for optimal benefit from consolidative chemoradiation therapy. Cancer.

[3] Johung K, Saif MW, Chang BW (2012). Treatment of locally advanced pancreatic cancer: the role of radiation therapy. Int J Radiat Oncol Biol Phys.

[4] Iacobuzio-Donahue CA, Fu B, Yachida S, Luo M, Abe H, Henderson CM (2009). DPC4 gene status of the primary carcinoma correlates with patterns of failure in patients with pancreatic cancer. Journal of clinical oncology : official journal of the American Society of Clinical Oncology..

[5] Klaassen DJ, MacIntyre JM, Catton GE, Engstrom PF, Moertel CG (1985). Treatment of locally unresectable cancer of the stomach and pancreas: a randomized comparison of 5-fluorouracil alone with radiation plus concurrent and maintenance 5-fluorouracil--an eastern cooperative oncology group study. J Clin Oncol : official journal of the American Society of Clinical Oncology.

[6] Chauffert B, Mornex F, Bonnetain F, Rougier P, Mariette C, Bouche O (2008). Phase III trial comparing intensive induction chemoradiotherapy (60 Gy, infusional 5-FU and intermittent cisplatin) followed by maintenance gemcitabine with gemcitabine alone for locally advanced unresectable pancreatic cancer. Definitive results of the 2000-01 FFCD/SFRO study. Annals of oncology : official journal of the European society for. Med Oncol.

[7] Loehrer PJ, Feng Y, Cardenes H, Wagner L, Brell JM, Cella D (2011). Gemcitabine alone versus gemcitabine plus radiotherapy in patients with locally advanced pancreatic cancer: an eastern cooperative oncology group trial. Journal of clinical oncology : official journal of the American Society of Clinical Oncology.

[8] Hammel P, Huguet F, van Laethem JL, Goldstein D, Glimelius B, Artru P (2016). Effect of Chemoradiotherapy vs chemotherapy on survival in patients with locally advanced pancreatic Cancer controlled after 4 months of gemcitabine with or without Erlotinib: the LAP07 randomized clinical trial. JAMA.

[9] Conroy T, Desseigne F, Ychou M, Bouche O, Guimbaud R, Becouarn Y (2011). FOLFIRINOX versus gemcitabine for metastatic pancreatic cancer. N Engl J Med.

[10] Suker M, Beumer BR, Sadot E, Marthey L, Faris JE, Mellon EA (2016). FOLFIRINOX for locally advanced pancreatic cancer: a systematic review and patient-level meta-analysis. The Lancet Oncology.

[11] Von Hoff DD, Ervin T, Arena FP, Chiorean EG, Infante J, Moore M (2013). Increased survival in pancreatic cancer with nab-paclitaxel plus gemcitabine. N Engl J Med.

[12] Nakamura A, Shibuya K, Matsuo Y, Nakamura M, Shiinoki T, Mizowaki T (2012). Analysis of dosimetric parameters associated with acute gastrointestinal toxicity and upper gastrointestinal bleeding in locally advanced pancreatic cancer patients treated with gemcitabine-based concurrent chemoradiotherapy. Int J Radiat Oncol Biol Phys.

[13] Yovino S, Poppe M, Jabbour S, David V, Garofalo M, Pandya N (2011). Intensity-modulated radiation therapy significantly improves acute gastrointestinal toxicity in pancreatic and ampullary cancers. Int J Radiat Oncol Biol Phys.

[14] Colbert LE, Moningi S, Chadha A, Amer A, Lee Y, Wolff RA (2017). Dose escalation with an IMRT technique in 15 to 28 fractions is better tolerated than standard doses of 3DCRT for LAPC. Advances in radiation oncology.

[15] Oya N, Shibuya K, Sakamoto T, Mizowaki T (2006). R, Fujimoto K, et al. Chemoradiotherapy in patients with pancreatic carcinoma: phase-I study with a fixed radiation dose and escalating doses of weekly gemcitabine. Pancreatology : official journal of the international association of. Pancreatology.

[16] Shibuya K, Oya N, Fujii T (2011). R, Nakamura a, Matsuo Y, et al. phase II study of radiation therapy combined with weekly low-dose gemcitabine for locally advanced, unresectable pancreatic cancer. Am J Clin Oncol.

[17] Murphy JD, Adusumilli S, Griffith KA, Ray ME, Zalupski MM, Lawrence TS (2007). Full-dose gemcitabine and concurrent radiotherapy for unresectable pancreatic cancer. Int J Radiat Oncol Biol Phys.

[18] Bittner MI, Grosu AL, Brunner TB (2015). Comparison of toxicity after IMRT and 3D-conformal radiotherapy for patients with pancreatic cancer - a systematic review. Radiotherapy and oncology : journal of the European Society for Therapeutic Radiology and Oncology.

[19] Ben-Josef E, Schipper M, Francis IR, Hadley S, Ten-Haken R, Lawrence T (2012). A phase I/II trial of intensity modulated radiation (IMRT) dose escalation with concurrent fixed-dose rate gemcitabine (FDR-G) in patients with unresectable pancreatic cancer. Int J Radiat Oncol Biol Phys.

[20] Krishnan S, Chadha AS, Suh Y, Chen HC, Rao A, Das P (2016). Focal radiation therapy dose escalation improves overall survival in locally advanced pancreatic Cancer patients receiving induction chemotherapy and consolidative Chemoradiation. Int J Radiat Oncol Biol Phys.

